# Leveraging existing market incentives to increase HIV pre‐exposure prophylaxis access in the United States

**DOI:** 10.1002/jia2.26504

**Published:** 2025-07-02

**Authors:** Jirair Ratevosian, Caroline Piselli, Patrick Sullivan, LaRon E. Nelson

**Affiliations:** ^1^ Yale School of Nursing Yale University New Haven Connecticut USA; ^2^ Center for Interdisciplinary Research on AIDS Yale University New Haven Connecticut USA; ^3^ Rollins School of Public Health Emory University Atlanta Georgia USA; ^4^ Yale School of Public Health Yale University New Haven Connecticut USA; ^5^ University of Toronto Rotman School of Management Toronto Ontario Canada

1

Pre‐exposure prophylaxis (PrEP) is a highly effective tool in the response to end the HIV epidemic, reducing transmission risk when taken consistently [1]. First approved by the U.S. Food and Drug Administration in 2012, PrEP has become a cornerstone of HIV initiatives in the United States. In 2025, a key policy change to improve the financial incentives for health insurers to cover PrEP could significantly boost access to HIV prevention, bringing national health financing policy in line with public health priorities.

Inadequate access to and substantial inequities in uptake are currently limiting the full positive impacts of PrEP on the health of Americans [2]. In 2023, only approximately one‐third of people who would benefit from PrEP were using it [3]. Further, the patterns of PrEP usage were not always trending towards the populations with the highest likelihoods of exposure to HIV. For example, PrEP use was not proportionate to the risk of HIV for women, Black and Hispanic communities, and adolescents [2]. These inequities in PrEP use are attributable to many factors, including lower coverage of health insurance for these groups and a lack of local policies that result in high out‐of‐pocket costs for those without insurance coverage for PrEP [4, 5].

According to the U.S. Census Bureau, in 2023, approximately 92.0% of Americans had health insurance coverage at some point during the year. Private health insurance was more prevalent, covering 65.4% of the population, primarily through employer‐sponsored plans (53.7%). Public insurance programmes, including Medicare, Medicaid and the Veterans Health Administration, covered 36.3% of individuals [6]. These gains in coverage are largely attributable to the Affordable Care Act (ACA), which became law in 2010. The ACA introduced insurance marketplaces, expanded Medicaid eligibility and mandated coverage of essential health benefits, including preventive services.

The ACA also requires coverage for preventive services, including PrEP since 2021. Yet, insurers have traditionally imposed cost‐related policies that limited access to the medication, and many also view PrEP medication and related services, like routine lab tests and provider visits, as financial liabilities that increase their costs relative to the reimbursements they may receive from the government. As a result, individuals with high HIV exposure probabilities frequently encounter high insurance deductibles, limited provider networks and burdensome prior authorizations that impede PrEP access [7, 8].

According to a recent analysis, 13% of private U.S. insurance plans in 2024 did not list PrEP as no‐cost to enrolees in their prescription drug formularies, 31% did not list PrEP in their no‐cost preventive services list and 66% failed to clearly indicate whether essential services were covered without cost‐sharing by the enrolee [9]. These hurdles leave many insurance enrolees uncertain about their eligibility for no‐cost PrEP.

HPTN 096 is a study with strategic U.S. public health significance that is testing the efficacy of an integrated strategy to improve PrEP use among Black men in the American South. Data from the formative phase of the trial revealed a critical underlying issue: insurance companies were balancing a legal mandate to provide no‐cost PrEP with corporate mandates to meet earnings expectations. This dynamic disincentivizes insurers from promoting the use of PrEP and from enrolling individuals who would be routine users of the covered benefit—especially as higher‐cost PrEP options come online. This gap exposed a policy misalignment undermining national HIV prevention goals.

Under the ACA, all U.S. insurers are required to cover U.S. Preventive Services Task Force (USPSTF) Grade A preventive services without cost sharing, which includes PrEP. However, the law does not mandate coverage for every PrEP formulation. As a result, while oral PrEP (e.g. generic tenofovir disoproxil fumarate and emtricitabine) must be covered at no cost, access to newer, more expensive options like long‐acting injectables depends on individual plan formularies and state‐level guidance. Insurers may still impose utilization management tools such as prior authorization or require patients to try cheaper options first, (for which inadequate compliance could lead to HIV)—a practice known as step therapy.

When the ACA became law, it introduced risk adjustment to stabilize insurance markets and to ensure that insurers cover high‐cost conditions like HIV. The ACA compensates insurers with higher clinical‐economic risk enrolees by transferring funds from those with lower costs. However, this did not apply to preventive services—so insurers that cover HIV prevention end up shouldering the full cost. This misalignment incentivizes restricting access to prevention rather than expanding it, despite its proven benefits in saving lives and reducing healthcare costs.

By 2024, mounting evidence and advocacy efforts pushed U.S. Centers for Medicare & Medicaid Services (CMS), the agency that oversees public health insurance programmes, to reconsider how PrEP might fit within the risk adjustment programme. The decision to reassess PrEP's inclusion marked a critical step towards aligning financial incentives with public health priorities. In 2025, CMS published new criteria to capture the costs associated with PrEP utilization among insurance enrolees, paving the way for the inclusion of HIV prevention in the risk adjustment programme [10].

In expanding risk adjustment to include PrEP, the U.S. Department of Health and Human Services outlined seven key principles for associated costs, including clinical relevance, predictable costs and a sufficient sample size, while also acknowledging time‐value considerations for insurers. Importantly, by recognizing PrEP as a critical preventive service, this policy shift aligns clinical recommendations [11] with business incentives for insurers to expand coverage.

Under this new policy, U.S. insurers offering PrEP services will be better positioned to offset the financial risks associated with enrolees that use these services. This shift is expected to reduce barriers to access and increase PrEP uptake, particularly in communities historically underserved by the healthcare system. By preventing new HIV acquisitions, broader PrEP access can also reduce the substantial long‐term healthcare costs directly associated with HIV treatment, which exceed U.S. $420,000 per person over a lifetime [12].

The U.S. experience highlights how financial misalignment between insurers and public health priorities can hinder HIV prevention efforts—a lesson relevant for countries navigating similar dynamics in expanding access to PrEP within insurance‐based systems. Although the inclusion of PrEP in the risk adjustment formula is a step in the right direction, much work remains to ensure full implementation by U.S. insurers. Potential government changes to HIV prevention funding could further reshape coverage [13]. Public health advocates should be vigilant to ensure the full implementation and to monitor any ongoing gaps in access for the people who need PrEP most.

## COMPETING INTERESTS

The authors declare no competing interests.

## AUTHORS’ CONTRIBUTIONS

JR conceptualized and drafted the article. CP, LEN and PS contributed substantial edits and revisions to the manuscript.

## Data Availability

Data sharing is not applicable to this article as no datasets were generated or analysed during the current study.
